# Decreased mortality in patients hospitalized due to respiratory diseases after installation of an intensive care unit in a secondary hospital in the interior of Brazil

**DOI:** 10.5935/0103-507X.20150043

**Published:** 2015

**Authors:** Luciano Passamani Diogo, Laura Fuchs Bahlis, André Wajner, Fernando Starosta Waldemar

**Affiliations:** 1 Hospital de Clínicas de Porto Alegre - Porto Alegre (RS), Brazil.; 2 Universidade Federal do Rio Grande do Sul - Porto Alegre (RS), Brazil.; 3 Hospital Medicine Service, Hospital Montenegro - Montenegro (RS), Brazil.

**Keywords:** Inpatients, Respiratory tract diseases/mortality, Secondary care centers, Intensive care units

## Abstract

**Objective:**

To evaluate the association between the in-hospital mortality of patients hospitalized due to respiratory diseases and the availability of intensive care units.

**Methods:**

This retrospective cohort study evaluated a database from a hospital medicine service involving patients hospitalized due to respiratory non-terminal diseases. Data on clinical characteristics and risk factors associated with mortality, such as Charlson score and length of hospital stay, were collected. The following analyses were performed: univariate analysis with simple stratification using the Mantel Haenszel test, chi squared test, *Student’s t* test, Mann-Whitney test, and logistic regression.

**Results:**

Three hundred thirteen patients were selected, including 98 (31.3%) before installation of the intensive care unit and 215 (68.7%) after installation of the intensive care unit. No significant differences in the clinical and anthropometric characteristics or risk factors were observed between the groups. The mortality rate was 18/95 (18.9%) before the installation of the intensive care unit and 21/206 (10.2%) after the installation of the intensive care unit. Logistic regression analysis indicated that the probability of death after the installation of the intensive care unit decreased by 58% (OR: 0.42; 95%CI 0.205 -0.879; p = 0.021).

**Conclusion:**

Considering the limitations of the study, the results suggest a benefit, with a decrease of one death per every 11 patients treated for respiratory diseases after the installation of an intensive care unit in our hospital. The results corroborate the benefits of the implementation of intensive care units in secondary hospitals.

## Abstract (pt)

**Objetivo:**

Avaliar relação entre a mortalidade intrahospitalar de pacientes internados por doenças respiratórias e a disponibilidade de unidades de terapia intensiva.

**Métodos:**

Foi realizada coorte retrospectiva do banco de dados em um serviço de medicina hospitalar. Selecionaram-se pacientes internados por doenças respiratórias não terminais. Características clínicas, fatores de risco associado à mortalidade, como o escore de Charlson, e tempo de internação foram coletados. Foram realizados: análise univariada com estratificação simples por Mantel Haenszel, e testes qui quadrado, *t* de *Student* e Mann-Whitney, além de regressão logística.

**Resultados:**

Foram selecionados 313 pacientes, 98 (31,3%) antes da instalação da unidade de terapia intensiva e 215 (68,7%) após a disponibilização de unidade de terapia intensiva. Quando comparados quanto a características clínicas, antropométricas e fatores de risco, não houve diferença significativa. A mortalidade antes da disponibilidade da unidade de terapia intensiva foi de 18/95 (18,9%) e, após, de 21/206 (10,2%). Na regressão logística, a chance de morte após implantação da unidade de terapia intensiva diminuiu em 58% (OR: 0,42; IC95% 0,205 - 0,879; p = 0,021).

**Conclusão:**

Respeitando as limitações do estudo, conjetura-se benefício na redução de uma morte a cada 11 pacientes tratados por doenças respiratórias após a implantação da unidade de terapia intensiva no hospital. Estes resultados corroboram a impressão do benefício da implantação de unidades de terapia intensiva em hospitais de nível secundário.

## INTRODUCTION

Increased healthcare costs have been a cause for concern for governments and the private sector. In the public health system - Unified Health System (Sistema Único de Saúde - SUS) in the case of Brazil - this concern is greater owing to the high demands of health users and the limited availability of resources. A World Bank study investigated the 20-year implementation of SUS by the federal government and reported that at present, Brazil spends approximately 4% of its Gross Domestic Product (GDP) on healthcare and that since the early 2000s, this number has increased approximately 6% per year.^(1)^ Furthermore, there is a worldwide trend of population aging. In Brazil, the percentage of citizens aged > 60 years increased from 6% to 10% between 1980 and 2010 and is expected to reach nearly 30% by 2050.^(2)^ Therefore, the tendency is that these costs will increase even further in the coming years, and these costs will include the implementation of intensive care units (ICU).^(3)^ In parallel, the costs related to the installation and maintenance of ICU have reportedly increased.^(4,5)^

Intensive care units are implemented because of advancements in technology and the therapeutic arsenal. The patients assisted in these units include those in vulnerable conditions with limited physiological function, severe illnesses, and illnesses of complex management. The need for ICU implementation is clear, and the ability of ICU to decrease morbidity and mortality in the hospital setting has not been questioned. Perhaps for this reason, only a few studies have evaluated the impact of ICU on morbidity and mortality. Previous studies have reported increased mortality in subgroups of patients not admitted to ICU because of the unavailability of beds.^(6-8)^ Other studies involving patients admitted to stroke treatment units indicated decreased morbidity and mortality and decreased length of stay in this specific population.^(9-12)^

Taking into account the exponential increase in financial investments for the installation and maintenance of ICU^(4)^ and the lack of evidence demonstrating their direct benefits in all contexts, questions arise concerning the cost-benefit of their implementation. The clarification of where and when ICU should be implemented and their degree of complexity is necessary to evaluate their cost-benefit in a particular region^(13,14)^ and is essential for the development of public policies.^(14)^

To contribute to the understanding of the true benefits of ICU in hospital settings, we evaluated the impact of the installation of an ICU on the mortality of patients hospitalized due to respiratory diseases in a medical ward of a secondary public hospital in the interior of the state of Rio Grande do Sul. Respiratory diseases were chosen as the focus of this study because they are the leading cause of hospitalization in the evaluated hospital. In addition, according to internal data of our medical service, patients with respiratory diseases are at increased risk of mortality compared with other diseases.

## METHODS

This uncontrolled before and after study analyzed a hospital medicine service database and was performed prospectively in *Hospital Montenegro*. Located in Montenegro, approximately 50 km from the capital of Rio Grande do Sul state, the *Hospital Montenegro* has a coverage of 19 municipalities, totaling approximately 160,000 inhabitants. The hospital is of medium complexity (secondary level), with 150 beds, and provides services only to SUS. The ICU has 10 beds, and all beds exhibit multi-parameter monitoring and the availability of mechanical ventilators - both invasive and non-invasive. The healthcare team consisted of two routine intensive care physicians working for a daily period of 12 hours, a ratio of one nursing technician per 1.3 beds, and two nurses per shift. The physical therapy team was present in daily shifts of 18 hours. The study was approved by the Research Ethics Committee of the *Hospital de Clínicas de Porto Alegre*, and the need for free and informed consent was waived.

The study included patients aged 18 years and older who were hospitalized due to respiratory system diseases (exacerbated chronic obstructive pulmonary diseases (COPD), pneumonia and decompensated asthma). Patients considered terminal on admission were excluded.

Data collection was performed between May 2013 and June 2014 by trained staff and was reviewed by the healthcare team.^(15)^ The clinical and demographic characteristics of the patients were recorded, including age, sex, ethnicity, education, place of residence (urban or rural), presence of ICU during hospital stay, pathology that led to hospitalization, Charlson severity score,^(16)^ length of stay, and hospital outcome (discharge, hospital transfer, or death).

### Statistical analysis

For a mortality rate of 20% before the installation of the ICU and 10% after the installation of the ICU, a significance level of 5%, and a power of 80%, the sample size needed for this cohort was calculated to be 312 patients.

Descriptive statistics were used to analyze the incidence of hospitalization due to the aforementioned conditions as well as demographic and social characteristics. Comparisons were performed between the outcomes and participants’ characteristics before and after the installation of the ICU. Continuous and normally distributed variables are expressed using means and are compared using *Student’s* *t*-test for unpaired samples. The variables that did not meet the criteria for normality are described by median and compared using the Mann-Whitney test. Dichotomous variables are compared using the chi-square test or Fischer’s exact test, according to their distribution (a dichotomous variable was created for description of the periods before and after installation of the ICU). Univariate analysis was performed by simple stratification using the Mantel-Haenszel test, and variables with ≤ 0.1 were selected for analysis using logistic regression (backward stepwise) with death as the primary outcome.

Data were entered into Excel^®^ and, after review, were analyzed using Statistical Package for Social Sciences (SPSS) and Epi Info 7 software.

## RESULTS

Between May 2013 and June 2014, 313 patients who met the inclusion criteria were selected: 98 (31.3%) and 215 (68.7%) patients before and after installation of the ICU, respectively. Twelve patients were excluded from the final analysis because their condition was terminal, including 3 before the installation of the ICU and 9 after the installation of the ICU. Therefore, the final analysis included 301 patients. The incidence of respiratory illnesses among the participants was 24.84% (313 patients from a total of 1,260 admissions to hospital medical services during the study period).

No significant differences were observed with respect to the clinical characteristics, anthropometric characteristics, or risk factors between the study groups before and after the installation of the ICU, as shown in table 1.

**Table 1:** Characteristics of the study groups

**Variable**	**With ICU N = 206**	**Without ICU N = 95**	**p value**
Age	67 ± 17	66 ± 18	0.740
Age > 60 years	143 (69.4)	59 (62)	0.209
Male	118 (57.3)	56 (58.9)	0.78
Charlson score	2.22 ± 2.28	2.15 ± 1.17	0.568
Charlson score > 3	41 (19.9)	16 (16.8)	0.529
Length of hospital stay	8.27 ± 9.74	7.07 ± 8.04	0.689
Length of stay (days)†	5 (3 - 10)	5 (3 - 9.25)	0.686
Cancer	16 (7.8)	5 (5.3)	0.428
Dialysis	1 (0.5)	0	0.173
Length of hospital stay > 10 days	47 (22.8)	20 (21.1)	0.733
Hospital transfer	3 (1.5)	3 (3.2)	0.326
Multidrug-resistant pathogens	26 (12.6)	7 (7.4)	0.241
Use of antibiotics	193 (93.7)	89 (93.7)	0.999
Origin			
From our hospital‡	179 (86.9)	15 (75)	0.145
From other hospitals‡	9 (4.4)	1 (5)	0.890
Residence in Montenegro	142 (68.9)	70 (73.3)	0.401

ICU - intensive care unit. Mann-Whitney U test; Pearson chi-square test or Fisher's exact test;

† median and interquartile range;

‡ total sample size = 226. Results are expressed as the mean ± standard deviation and relative number (percentage).

Among the 206 patients with respiratory diseases admitted to the ward after the installation of the ICU, 40 (19.43%) remained in the ICU itself; 9 of these patients (22.5%) died. Twelve patients died in the regular hospitalization unit; 2 patients died after assistance by the emergency staff but before admission to the ICU, and 3 patients suffered cardiac arrest or death in the regular admission unit. The medical staff and family established that the remaining 7 patients would no longer benefit from ICU care during the hospitalization period after multiple treatment attempts (some were admitted to the ICU, but their clinical condition worsened).

Approximately 57% of cases (23 patients) of ICU admissions occurred in less than 24 hours of hospital admission, whereas the remaining 43% cases (17 patients) remained at least 2 days in the ward, with an indication of transfer to the ICU after this period. There were no reports of delayed ICU admission secondary to exceeded ICU capacity during the study period. Hospital transfer occurred for 3 (3.2%) patients before the installation of the ICU and for 3 (1.5%) patients after the installation of the ICU (p = 0.326).

In the univariate analysis, the probability of death after the installation of the ICU decreased by 52% (odds ratio - OR: 0.48; 95% confidence interval - 95%CI 0.24 - 0.96; p = 0.036). Logistic regression analysis indicated a decrease of 57.5% (OR: 0.425; IC95% 0.205 - 0.879; p = 0.021), as shown in table 2. The calculated number necessary for treatment was 10.43.

**Table 2:** Results of the final logistic regression model for hospital mortality

**Variable**	**OR**	**95%CI**	p value*
Presence of ICU in the hospital	0.425	0.205 - 0.879	0.021
Charlson score > 3	2.718	1.253 - 5.890	0.011
Length of hospital stay > 10 days	3.770	1.800 - 7.860	< 0.001

OR - odds ratio; 95%CI - 95% confidence interval; UTI - intensive care unit.

* Variables that were entered Step 1: presence or absence of ICU, Charlson score > 3, age > 60 years, presence of non-terminal cancer, hospitalization > 10 days, need to undergo any surgical procedure, residence in rural areas, and infection with multidrug-resistant pathogens.

## DISCUSSION

Previous studies have reported the benefits of the implementation of specific healthcare units.^(10,12,17)^ However, there is a gap in the literature with respect to ICUs.^(14)^ Therefore, this study attempted to address this gap and successfully demonstrated the positive results of creating an ICU in a medium-sized hospital. Of note is the number necessary for treatment of 10.43, i.e., the estimation that one death can be avoided for every 10.43 admissions due to respiratory diseases.

This study was possible because of the existence of records of clinical and demographic characteristics and of the degree of severity (such as the Charlson score) before the installation of the ICU, permitting the assessment of the performance of the hospital medical staff and the evaluation of the risk factors associated with unfavorable outcomes. Interestingly, in the period before the installation of the ICU, the patients with respiratory diseases exhibited an increased risk of mortality compared with other patient groups. During this period, patients admitted due to respiratory diseases were assisted in the ward or were allocated to emergency rooms, where they received care by the unit staff. Only three patients were transferred to other institutions with ICU.

After the installation of ICU, a Charlson score of > 3 points and prolonged length of stay remained as significant risk factors for mortality in our hospital, whereas admission due to respiratory disease was no longer a risk factor, reinforcing our findings.

The impact of the installation of an ICU on the decrease of mortality was extremely significant for respiratory diseases. Although the effect of seasonality on patient mortality is possible, no differences were observed in the severity score of patients when the pre- and post-installation periods were compared. In addition, the winter period was evaluated both before and after installation of the ICU.

One of the limitations of the study includes its design. Uncontrolled before and after studies are susceptible to biases related to temporality. However, in contrast to quasi-experimental studies, the Hawthorne effect does not occur.^(18)^ In our hospital, during the study period, there was no structural improvement other than the installation of the ICU. However, to our understanding, these measures could improve the quality of care and the management of this population and therefore justify the results obtained after implantation of the ICU. Furthermore, no changes were made in the medical staff in the inpatient unit or in the nursing or physical therapy staff. Despite the retrospective nature of this study, data were collected prospectively with a well-defined goal, as described above. Furthermore, the outcome of interest, i.e., mortality, is resistant to collection error. Another limitation was related to the smaller number of patients before the implantation of the ICU relative to after installation. The smaller number of patients before the installation of the ICU was due to the date at which systemic data collection in the hospital medicine service was initiated (only three months before the installation of the ICU).

Despite the demonstrated similarity between the study groups, it is possible that the degree of severity of the patients before and after the installation of the ICU may have differed. It is possible that for some reason, the patients before the installation were more severely ill, leading to increased mortality in this group. The most appropriate strategy to compare the groups would be to assign disease severity scores using the Sequential Organ Failure Assessment (SOFA) or the Acute Physiology and Chronic Health Evaluation (APACHE). However, before the installation of the ICU, no data were collected on these scores, which typically have their use restricted to the ICU. Therefore, the Charlson score was chosen as the method to compared disease severity between the two groups. This score is well validated in the literature as a predictor of in-hospital mortality in many situations, including respiratory diseases.^(19-21)^ The mean score was similar in both the groups, which reinforces the fact that our results were associated with the installation of the ICU.

Our study is the first to demonstrate the actual effect of installation of an ICU in a secondary public hospital in Brazil. Although it is impossible to think of a tertiary hospital without an ICU, because of the lack of evidence to test this hypothesis (as is often the case in situations where the benefit is obvious),^(22)^ the cost-benefit of these units in smaller hospitals can be questioned.

## CONCLUSION

This study is the first to demonstrate the benefits of implementing an intensive care unit in a secondary hospital in Brazil. Only patients with respiratory diseases were considered in this study. Therefore, additional studies are needed to assess the impact of these units on other patient groups.
